# Sub-2 nm Thick Fluoroalkylsilane Self-Assembled Monolayer-Coated High Voltage Spinel Crystals as Promising Cathode Materials for Lithium Ion Batteries

**DOI:** 10.1038/srep31999

**Published:** 2016-08-24

**Authors:** Nobuyuki Zettsu, Satoru Kida, Shuhei Uchida, Katsuya Teshima

**Affiliations:** 1 Center for Energy and Environmental Science, Shinshu University, 4-17-1 Wakasato, Nagano 380-8553, Japan; 2 Department of Materials Chemistry, Shinshu University, 4-17-1 Wakasato, Nagano 380-8553, Japan

## Abstract

We demonstrate herein that an ultra-thin fluoroalkylsilane self-assembled monolayer coating can be used as a modifying agent at LiNi_0.5_Mn_1.5_O_4−*δ*_cathode/electrolyte interfaces in 5V-class lithium-ion batteries. Bare LiNi_0.5_Mn_1.5_O_4−*δ*_ cathode showed substantial capacity fading, with capacity dropping to 79% of the original capacity after 100 cycles at a rate of 1C, which was entirely due to dissolution of Mn^3+^ from the spinel lattice via oxidative decomposition of the organic electrolyte. Capacity retention was improved to 97% on coating ultra-thin FAS17-SAM onto the LiNi_0.5_Mn_1.5_O_4_ cathode surface. Such surface protection with highly ordered fluoroalkyl chains insulated the cathode from direct contact with the organic electrolyte and led to increased tolerance to HF.

High-voltage spinel (LiNi_0.5_Mn_1.5_O_4_) is considered one of the most promising cathode materials for use in Li-ion batteries for hybrid electric vehicles (HEVs) and plug-in hybrid electric vehicles (PHEVs) due to its high voltage plateau, at around 4.7 V. This results in its energy density (650 Wh·kg^−1^) being over 20% higher than those of conventional LiCoO_2_-, LiMn_2_O_4_-, and LiFePO_4_-based materials. However, the working potential of LiNi_0.5_Mn_1.5_O_4_ approaches the thermodynamic stability limit of carbonate-based electrolytes; hence, a systematic research approach is needed to enhance the compatibility of LiNi_0.5_Mn_1.5_O_4_ with electrolytes to improve its cycle life and safety characteristics^1^,^2^,^3^.

It is well established that the presence of trace amounts of HF in the non-aqueous electrolyte plays an important role in cathode corrosion, which decreases cell life. Reactions with cathode materials tend to be accelerated on increasing the temperature and cut-off voltages^4^,^5^,^6^. A common approach to tackling this corrosion has been the application of a surface coating to the cathode material that functions as an HF scavenger to locally neutralise the electrolyte. Coatings that have been applied in previous research, such as Li_3_PO_4_^7^, AlPO_4_^8^, ZnO_*x*_^9^, LiFePO_4_^10^, graphene nanosheets^11^, and NbO_*x*_ nanosheets^12^, act as a physical barrier that prevents direct contact between the cathode material and electrolyte. Surface coating has been consistently reported to improve the cycle life of lithium-ion cells, and even to allow materials to be cycled to higher working potentials for higher specific capacities with reasonable capacity retention. Surface coatings can be applied using several techniques, including sol-gel coating, chemical vapour deposition, co-precipitation, spin coating, and other physical deposition techniques^7^,^8^,^9^,^10^,^11^,^12^ These methods lead to a thin layer, but obtaining a complete coating necessary for the best possible protection is difficult, meaning that these coating techniques provide only limited improvements in cycle capability^2^.

This underlying problem with LiNi_0.5_Mn_1.5_O_4_ cathodes led to the use of an organosilane self-assembled monolayer (SAM) that homogeneously forms an ultra-thin yet dense layer on the oxide surface. It is known that SAMs are both mechanically and chemically stable due to their strong immobilisation on the oxide surface through the formation of siloxane bonds and the highly ordered structures of individual molecules, which are supported by intermolecular interactions^13^. Thus, SAMs have been frequently applied to modify the chemical and physical properties of solid surfaces. In this work, we studied the effects of sub-2 nm thick FAS-SAM coatings on the high voltage capability of the LiNi_0.5_Mn_1.5_O_4−*δ*_ cathode toward 5V-class lithium-ion batteries.

## Results and Discussion

As an example of an organosilane molecule, we used fluoroalkylsilane (FAS17: heptadecafluoro-1,1,2,2-tetrahydro-decyl-1-trimethoxysilane, F_3_C(CF_2_)_7_(CH_2_)_3_Si(OCH_3_)_3_, which is known to be mechanically and electrochemically stable^14^,^15^. Although liquid-phase processing is usually used for SAM preparation, we applied a vapour-phase process^16^ because it was expected to reduce the deposition of aggregated organosilane molecules, which tend to degrade the quality of SAMs. The method employed herein resulted in the formation of a homogenous, fully coated ultra-thin layer on the LiNi_0.5_Mn_1.5_O_4−*δ*_ surface.

**Figure 1:**
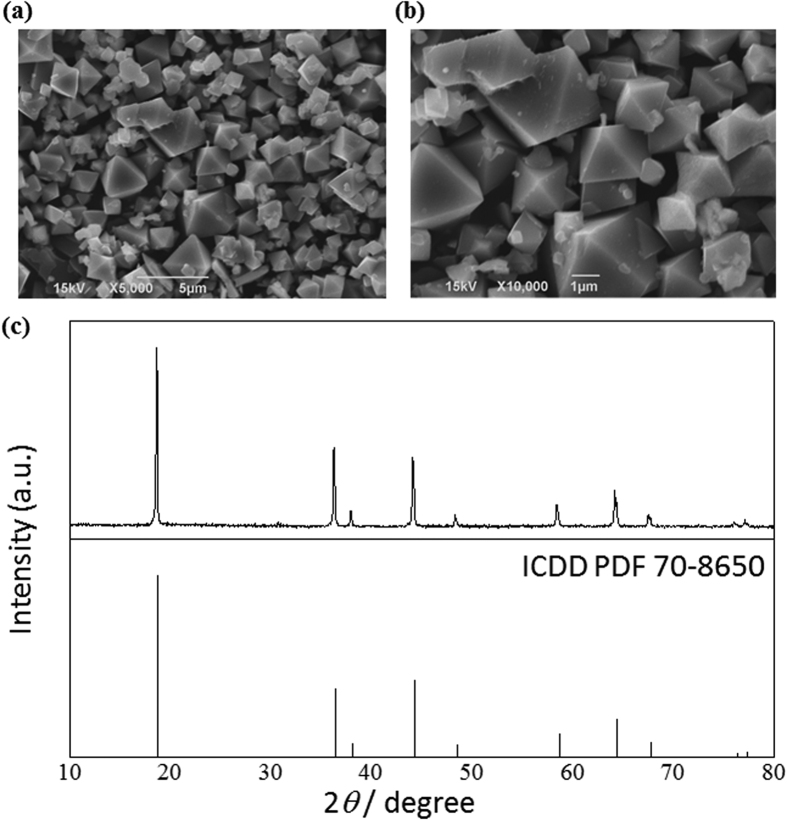
(**a,b**) SEM images and (**c**) powder XRD pattern of LiNi_0.5_Mn_1.5_O_4−*δ*_ crystals grown from a LiCl-KCl flux.

The LiNi_0.5_Mn_1.5_O_4−*δ*_ crystals used were prepared by the flux growth method^17^,^18^,^19^,^20^,^21^,^22^. As shown in Fig. 1, field-emission scanning electron microscopy (FE-SEM) revealed octahedral crystals having well-defined facets, which were grown from a LiCl-KCl flux at 700 °C over 10 h. The powder X-ray diffraction pattern was in good agreement with the reference data (ICDD PDF 70-8650), indicating that the crystals formed the spinel structure. Raman spectroscopy further revealed that the ordering of Ni/Mn was random. Characteristic bands corresponding to the cation ordered structure were not observed clearly. Therefore, it is suggested that each element (Li/Ni/Mn/O) in the crystal was organised into an *Fd*-3m lattice. (see Fig. S1). The average diameter (D_50_), as evaluated using a particle size analyser, and the surface area, as derived from BET-based N_2_ absorption, were 1.01 μm and 0.32 m^2^·g^−1^, respectively. Self-assembly of FAS molecules at the vapour/LiNi_0.5_Mn_1.5_O_4−*δ*_ crystal surface was performed at 150 °C and atmospheric pressure.

No detectable changes were observed in the XRD pattern or morphology of the crystal after the FAS17-SAM coating procedure. Only the chemical environments of the LiNi_0.5_Mn_1.5_O_4−*δ*_ crystal surface were changed, which was studied using X-ray photoelectron spectroscopy (XPS). A sharp peak at 688.8 eV assigned to the fluoroalkane group and a broad peak centred at 102.5 eV were observed in the XPS-F1s and -Si2p_2/3_ core-level spectra of the LiNi_0.5_Mn_1.5_O_4−*δ*_ crystal surface after treatment with FAS17-SAM (see Fig. S2a–c)^23^. The broad peak in the XPS-Si2p_2/3_ spectrum can be separated into three peaks by deconvolution analysis, with peaks at 101.9, 102.6, and 103.5 eV being attributed to Si-C, C-Si-O, and O-Si-C groups, respectively^16^,^24^. Furthermore, the peak area ratios of F1s/Mn2p_2/3_ and Si2p_2/3_/Mn2p_2/3_ drastically changed with increasing treatment time, and became constant after 15 h as shown in Fig. S2d,e. If multilayer inhomogeneous aggregates were formed, the relative intensities of these peaks would increase linearly with respect to time. This indicates that the LiNi_0.5_Mn_1.5_ O_4−*δ*_ surface was fully covered with a SAM of FAS17 molecules according to the Langmuir absorption model. Previous work by the Takai group found that the thickness of the FAS17-SAM formed on a SiO_2_/Si substrate was 1.34 nm. This value suggests that individual FAS molecules are closely packed in the monolayer and inclined >30° to normal^25^. We evaluated the thickness of the FAS-SAM coating layer experimentally through a combination of angle-resolved XPS-Mn2p and semi-empirical analysis (see Fig. S3)^26^,^27^,^28^. The sample thicknesses after 5 h and 15 h of treatment were 0.82 nm and 1.27 nm, respectively. The result agrees closely with the previous report, wherein the evaluation was carried out by ellipsometry.

**Figure 2:**
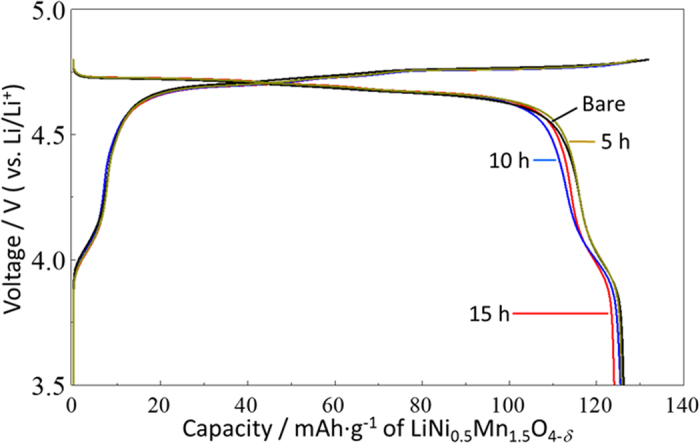
Galvanostatic charge-discharge curves of the FAS17-SAM-coated LiNi_0.5_Mn_1.5_O_4−*δ*_ cathode-based half-cells fabricated with different SAM coating times, collected at a rate of 0.2C between 3.5 and 4.8 V.

**Figure 3:**
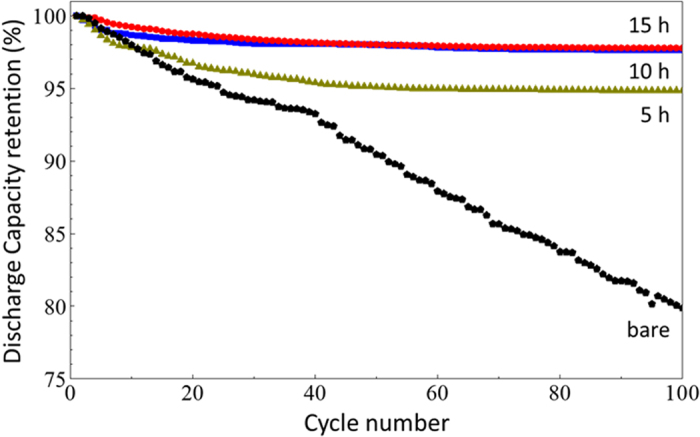
Cyclability of the FAS17-SAM-coated LiNi_0.5_Mn_1.5_O_4−*δ*_ cathode-based half-cells fabricated with different SAM coating times, collected at a rate of 1C between 3.5 and 4.8 V.

To evaluate the electrochemical properties of the FAS17-SAM-coated LiNi_0.5_Mn_1.5_O_4−*δ*_ composite electrodes composed of LiNi_0.5_Mn_1.5_O_4−*δ*_/acetylene black/PVDF = 90/5/5 (wt%), half-cells were assembled using lithium foil as a counter electrode. Figure 2 shows the galvanostatic charge-discharge curves of electrodes coated for 5, 10, and 15 h, with the curve of an uncoated electrode for comparison, after 3 cycles at 0.2 C (30 mA·g^−1^) and 20 °C. The horizontal axis was converted to mAh·g^−1^ of LiNi_0.5_Mn_1.5_O_4−*δ*_ crystals by removing the weight of the FAS-SAM layer from the total weight of the cathode. The capacity of the four different cells under the first three cycles is listed in Table S1. Note that no capacity degradation was observed in the galvanostatic charge-discharge characteristics, indicating that the FAS17-SAM had little effect on mitigating Li^+^ transfer at the electrode/electrolyte interface at 0.2C. However, the FAS17-SAM layer played an important role in the cyclability and Mn dissolution from the oxide surface. To provide direct evidence for electrode stabilisation, the electrochemical stability of LiNi_0.5_Mn_1.5_O_4−*δ*_ composite electrodes with and without protective layers were tested through cycle tests performed at 1C (150 mA·g^−1^) and 25 °C (Fig. 3). After 100 cycles, the capacity retention of the bare cathode cell had dropped to ca. 79%, while the FAS17-SAM-coated LiNi_0.5_Mn_1.5_O_4−*δ*_ electrodes maintained over 95% of their efficiencies. Furthermore, we found that the capacity retention depended on the coating time of the FAS17-SAM layer and reached a maximum of over 97%. The coverage ratio as well as complete/incomplete coating of the FAS-SAM layer would impact the difference in capacity fading in the cycling tests of samples with different coating times.

Capacity retention dropped abruptly in the bare LiNi_0.5_Mn_1.5_O_4−*δ*_ electrodes after 40 cycles. Note that a dramatic difference was observed on the separator surface after cycling. After disassembling the coin cells, the separator of the bare LiNi_0.5_Mn_1.5_O_4−*δ*_ electrode was black, while in the case of the FAS-SAM-coated electrode it was off-white. In addition, the batteries were reusable when the cell was reassembled after washing the electrode surface with ethylene carbonate and replacing the old separator with a new one. These results suggest that high voltage operation poses a risk for higher capacity fade due to impedance growth and short circuit formation caused by oxidative decomposition of electrolytes and Mn^3+^ dissolution during the cycles^2^,^4^,^5^,^6^.

In order to understand the effect of the FAS17-SAM coating on capacity fading, the structural and electrochemical changes were studied using XPS, electrochemical impedance spectroscopy (EIS), and cyclic voltammetry (CV). The changes in the relative atomic concentration of Mn^3+^/Mn^4+^, as evaluated after 100 cycles at 1C, are summarised in Table S2. Before cycling, the ratio of Mn^3+^ to Mn^4+^ in the LiNi_0.5_Mn_1.5_O_4−*δ*_ electrode was constant; however, the ratio drastically decreased in the presence of the FAS17-SAM coating after cycling. To the best of our knowledge, this result is the first example of the suppression of metal dissolution from an oxide cathode at high voltage operation (4.8 V vs Li^+^/Li) by a sub-2 nm coating of an ultra-thin SAM, instead of cathode particle stabilisation through conventional powder and thin film coating.

**Figure 4:**
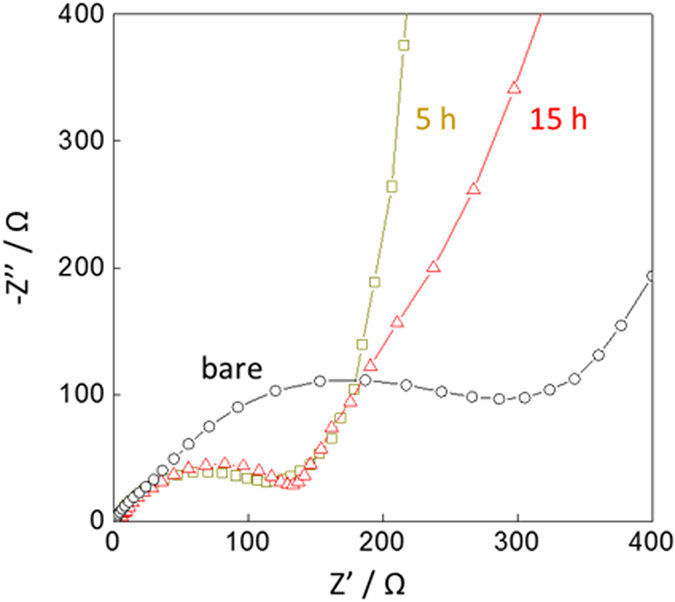
Nyquist plots of FAS17-SAM-coated LiNi_0.5_Mn_1.5_O_4−*δ*_ cathode-based half-cells fabricated with different SAM coating times, collected after the 100th cycle at a rate of 1C.

**Figure 5:**
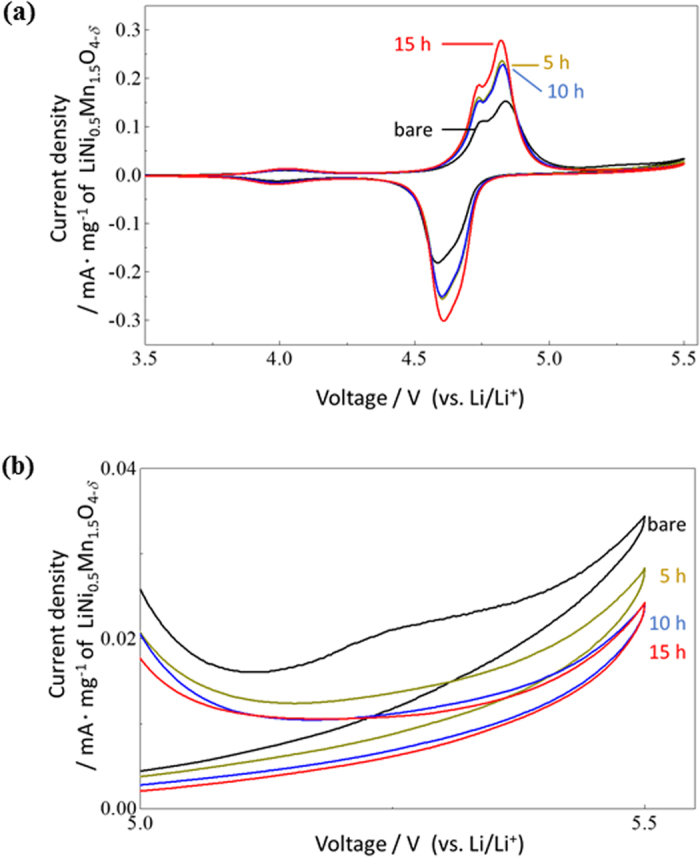
(**a**) Cyclic voltammograms of FAS17-SAM-coated LiNi_0.5_Mn_1.5_O_4−*δ*_ cathode-based half-cells fabricated with different SAM coating times, and (**b**) the corresponding extended voltammograms captured at 5.0–5.5 V.

The effect of the FAS-SAM on the impedance growth was also studied using EIS. A series of Nyquist plots obtained after 100 cycles at 3.5 V (vs Li^+^/Li) are shown in Fig. 4. A single semicircle corresponds to the charge transfer resistance at the interface of the electrode/electrolyte. The resistance decreased with increased amounts of FAS17-SAM coating. Under the experimental conditions used, the optimal resistance of the modified electrode was ca. 3 times smaller than for the unmodified electrode. F1s core level spectra taken from the LiNi_0.5_Mn_1.5_O_4−*δ*_ cathodes after cycling suggest that FAS17-SAM suppressed the formation of both LiF and MeF_2_ at the surface^9^. These chemicals do not conduct lithium ions well, leading to impedance growth. Furthermore, CV measurements revealed that the FAS17-SAM potentially insulated the LiNi_0.5_Mn_1.5_O_4−*δ*_ cathodes in the presence of a commonly used electrolyte containing LiPF_6_, which supressed the formation of LiF and MeF_2_. CV measurements were performed after 3 cycles under 0.2 C rate condition. As shown in Fig. 5, three distinct oxidative current peaks appeared at ca. 4.0, 4.6, and 4.7 V. These peaks can be assigned to oxidation of Mn^3+^/Mn^4+^, Ni^2+^/Ni^3+^, and Ni^3+^/Ni^4+^, respectively^2^. The FAS17-SAM coating amount had little effect on the peak position. Note that the bare LiNi_0.5_Mn_1.5_O_4−*δ*_ cathode exhibited a higher current density than the FAS17-SAM-coated LiNi_0.5_Mn_1.5_O_4−*δ*_ cathode at high voltages ranging from 5.0 to 5.5 V (vs. Li^+^/Li). The origin of the current density is the decomposition of the organic electrolyte at the electrode interface; thus, this result shows that FAS17-SAM prevented the interaction of the LiNi_0.5_Mn_1.5_O_4−*δ*_ surface with the electrolyte. Such interactions create a resistive solid–electrolyte interface on the cathode surface (cathode-SEI) and cause the degradation of the organic electrolyte, leading to capacity fading during cycling^2^,^9^.

**Figure 6:**
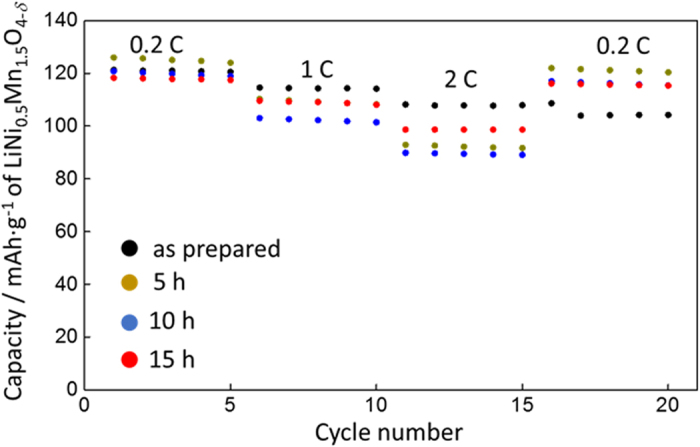
C rate capability of the FAS17-SAM-coated LiNi_0.5_Mn_1.5_O_4−*δ*_ cathode-based half-cells fabricated with different SAM coating times.

**Figure 7:**
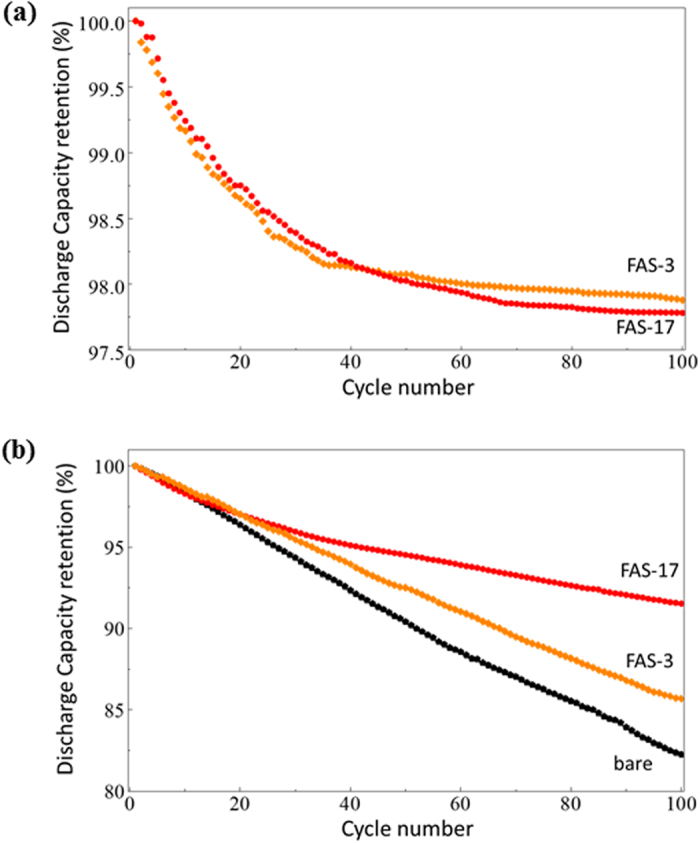
Cyclability of FAS3-SAM- and FAS17-SAM-coated LiNi_0.5_Mn_1.5_O_4−*δ*_ cathode-based half-cells, measured at (**a**) room temperature and (**b**) 55 °C.

It is interesting to note that the voltammograms also show that the FAS17-SAM coating enhanced the current densities corresponding to the redox reactions of Mn^3+^/Mn^4+^, Ni^2+^/Ni^3+^, and Ni^3+^/Ni^4+^. This result gives clear evidence that the highly ordered FAS17-SAM coating promoted the charge transfer reaction of the cathode and was responsible for the improved rate properties of the FAS17-SAM-coated LiNi_0.5_Mn_1.5_O_4_ cathodes. In fact, as shown in Fig. 6, the discharge capacities of the LiNi_0.5_Mn_1.5_O_4−*δ*_ cathodes with FAS17-SAM coating for 5 and 10 h decreased to less than 90 mAh·g^−1^ at 2C, while the LiNi_0.5_Mn_1.5_O_4−*δ*_ cathode coated with FAS17-SAM for 15 h still delivered over 100 mAh·g^−1^. The capacity retention was drastically increased in the LiNi_0.5_Mn_1.5_O_4−*δ*_ cathode coated with FAS17-SAM for 15 h, indicating that dense and highly ordered fluorocarbon chains are critical for improved rate properties. Although we are yet to obtain clear evidence, it is assumed that the highly ordered fluorocarbon chain allows for more rapid lithium insertion/extraction across the electrode/electrolyte interface through reduction of the desolvation energy of the lithium ion^29^,^30^. The replacement of the FAS17 molecules with FAS3, which has a shorter fluorocarbon chain, further demonstrated the effect of chain length (see Fig. 7). It was found that the use of FAS3 had no effect on the cycling capability at room temperature; however, it reduced cycle performance efficiency at 55 °C compared to that of FAS17. This indicates that both the concentration of surface fluoroalkyl groups and the stability of the closely packed SAM influence the cycle capability.

In summary, we have studied the impact of ultra-thin FAS-SAM coatings on LiNi_0.5_Mn_1.5_O_4−*δ*_ cathode surfaces on the high voltage capability of 5V-class lithium-ion batteries. It was revealed that a bare LiNi_0.5_Mn_1.5_O_4−*δ*_ cathode showed substantial capacity fading, with capacity dropping to 79% of the original capacity after 100 cycles at a rate of 1C, which was entirely due to dissolution of Mn^3+^ from the spinel lattice via oxidative decomposition of the organic electrolyte. Capacity retention was improved to 97% by coating an ultra-thin FAS17-SAM on the LiNi_0.5_Mn_1.5_O_4−*δ*_ cathode surface. Such surface protection with highly ordered fluoroalkyl chains insulated the cathode from direct contact with the organic electrolyte and led to increased tolerance to HF. Vapour-phase processing of FAS17-SAM at atmospheric pressure allowed the formation of a dense and homogenous coating of the protecting layer on the LiNi_0.5_Mn_1.5_O_4−*δ*_ cathode surface compared to conventional powder or thin film coating under vacuum. It should be noted that the thickness fraction of the FAS17-SAM was less than 0.1% (1.34 nm) compared to the mean diameter of the LiNi_0.5_Mn_1.5_O_4−*δ*_ crystal (1.01 μm); therefore the FAS17-SAM coating did not lose C rate capability due to increased charge transfer resistance. Furthermore, the C rate capability could be enhanced, which might be entirely due to reduction of the energy of desolvation of the lithium ion from the electrolyte because of the FAS17-SAM layer. It is concluded that SAMs containing fluoroalkane functional groups (e.g., FAS 17 and FAS 3) can be used as modifying agents at electrolyte/cathode interfaces in 5V-class lithium-ion batteries with a view to increasing their cycle capabilities without affecting their power densities.

## Method

### Flux growth of LiNi_0.5_Mn_1.5_O_4−*δ*_ crystals

The flux growth of LiNi0.5Mn_1.5_O_4−*δ*_ crystals was achieved from a stoichiometric mixture of Ni(NO_3_)_2_, Mn(NO_3_)_2_ and LiCl and using a binary flux of LiCl and KCl. All chemicals were purchased from Wako Pure Chemical Industries, Ltd. and were used without any additional purification. All powders were mixed in an alumina crucible with 30 mL volume. The solute concentration was controlled to be ca. 8 mol% for the flux growth reactions. The mixture was then heated to 700 °C in an electric furnace at a rate of 900 °C·h^−1^. After maintaining this temperature for 10 h, the crucible was cooled to 500 °C at a rate of 200 °C·h^−1^. The heated powders were then allowed to cool to room temperature naturally in the furnace. The powders were washed with warm water to remove the remaining flux. Finally, the powders were annealed under an O_2_ atmosphere at 700 °C for 10 h.

### FAS-SAM preparation

LiNi_0.5_Mn_1.5_O_4−*δ*_ crystals and a glass cup containing 0.2 cm^3^ FAS17 were placed in a 65 cm^3^ Teflon container. The container was sealed with a cap and placed in an oven maintained at 150 °C. FAS3-SAM was prepared under the same conditions using a different precursor molecule of F_3_C(CF_2_)_3_Si(OCH_3_)_3_ (Shin-etsu Chemical).

### Characterisation

The morphology of the as-grown LiNi_0.5_Mn_1.5_O_4−*δ*_ crystals was characterised using field-emission scanning electron microscopy (FE-SEM, JEOL, JSM-7600F) with an acceleration voltage of 15 kV. The phases and structures of the crystals were identified using XRD analysis with a Cu-Kα radiation source. The X-ray diffractometer (RIGAKU, MiniflexII) was operated at 30 kV and 20 mA, with 2θ = 10–80°. The chemical environments were analysed using X-ray photoelectron spectroscopy (XPS, JPS-9010, JEOL) with a monochromic Al source. All binding energies measured in XPS studies were referenced to the C1s hydrocarbon peak at 284.5 eV. The galvanostatic charge-discharge properties of all the electrodes were studied using a coin-type cell (R2032). The LiNi_0.5_Mn_1.5_O_4−*δ*_-based composite electrodes were prepared by a conventional pasting process and contained acetylene black (AB) and polyvinyldenefluoride (PVDF), which were added as electron conductivity and adhesion enhancement agents, respectively. The mixtures were diluted with N-methylpyrrolidone (NMP) to give a viscosity of 5.12 Pa·s. The prepared pastes were coated onto 20 μm thick Al foil using an applicator. The electrode density was adjusted to ca. 3.0 g·cm^−3^ using a roll press machine. The electrodes were dried under vacuum at 120 °C for 12 h prior to cell assembly. No significant changes were observed in the AB and PVDF after annealing. Lithium metal foil and polypropylene film were used as the counter electrode and separator, respectively. A solution of 1 M LiPF_6_ in ethylene carbonate (EC, C_3_H_4_O_3_)/dimethyl carbonate (DMC, C_3_H_6_O_3_) solution (EC:DMC = 3:7 v/v) was used as the electrolyte. A polypropylene separator (Celgard separator #2500) was used to suppress direct contact for each electrode. The coin-type lithium-ion batteries were assembled in an Ar-filled glove box (MIWA MFG Co. Ltd.) with a controlled atmosphere containing less than 1 ppm of H_2_O and O_2_. Galvanostatic charge-discharge tests and chemical impedance spectroscopy were conducted using a potentio/galvnostat (HOKUTO DENKO (HJ1001SD8) and Bio-Logic (VMP3)) under the designated conditions.

## Additional Information

**How to cite this article**: Zettsu, N. *et al*. Sub-2 nm Thick Fluoroalkylsilane Self-Assembled Monolayer-Coated High Voltage Spinel Crystals as Promising Cathode Materials for Lithium Ion Batteries. *Sci. Rep.* **6**, 31999; doi: 10.1038/srep31999 (2016).

## Supplementary Material

Supplementary Information
