# Supplementary material for: Sub-2 nm Thick Fluoroalkylsilane Self-Assembled Monolayer-Coated High Voltage Spinel Crystals as Promising Cathode Materials for Lithium Ion Batteries
Source: Sci Rep. 2016 Aug 24;6:31999. doi: 10.1038/srep31999 (PMC4995502; doi:10.1038/srep31999)
Supplement: Supplementary Information [file srep31999-s1.pdf]

Supplementary Information

Sub-2 nm Thick Fluoroalkylsilane Self-Assembled Monolayer-Coated High Voltage Spinel Crystals as Promising Cathode Materials for Lithium Ion Batteries

Nobuyuki Zettsu, Satoshi Kida, Shuhei Uchida, and Katsuya Teshima

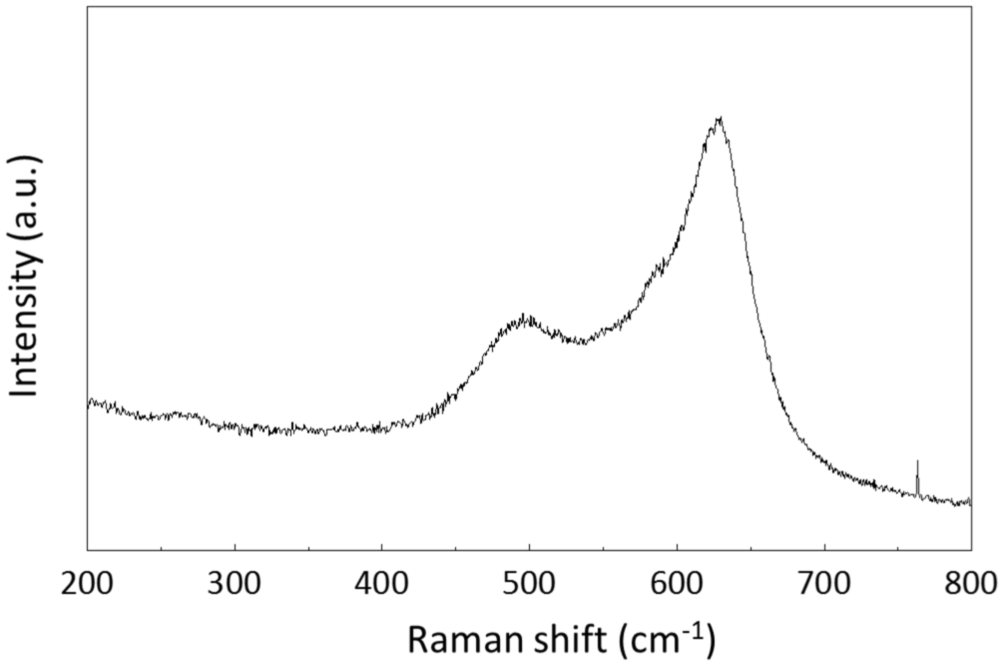

Fig. S1. Raman spectrum of as prepared $LiNi_{0.5}Mn_{1.5}O_{4-\delta}$ crystals grown from a LiCl-KCl molten flux.

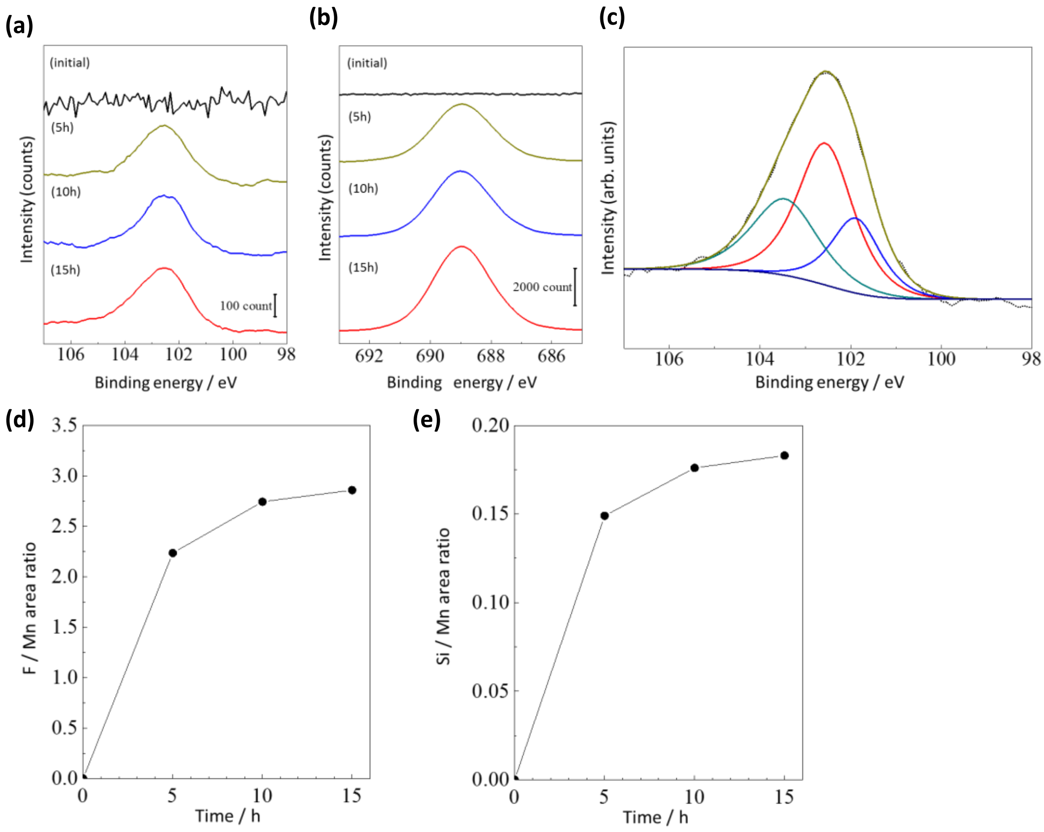

Fig. S2. (a) XPS F_{1s} and (b) Si_{2p} core level spectra of the FAS17-SAM-coated $LiNi_{0.5}Mn_{1.5}O_{4-\delta}$ crystals fabricated with different SAM coating times. (c) The peak deconvolution of the Si_{2p} core level spectra of the $LiNi_{0.5}Mn_{1.5}O_{4-\delta}$ substrate functionalized with FAS17-SAM. (d, e) Changes in the relative peak area of $F_{1s}/Mn_{2p2/3}$ (d) and $Si_{2p}/Mn_{2p2/3}$ (e) as a function of FAS-SAM coating time.

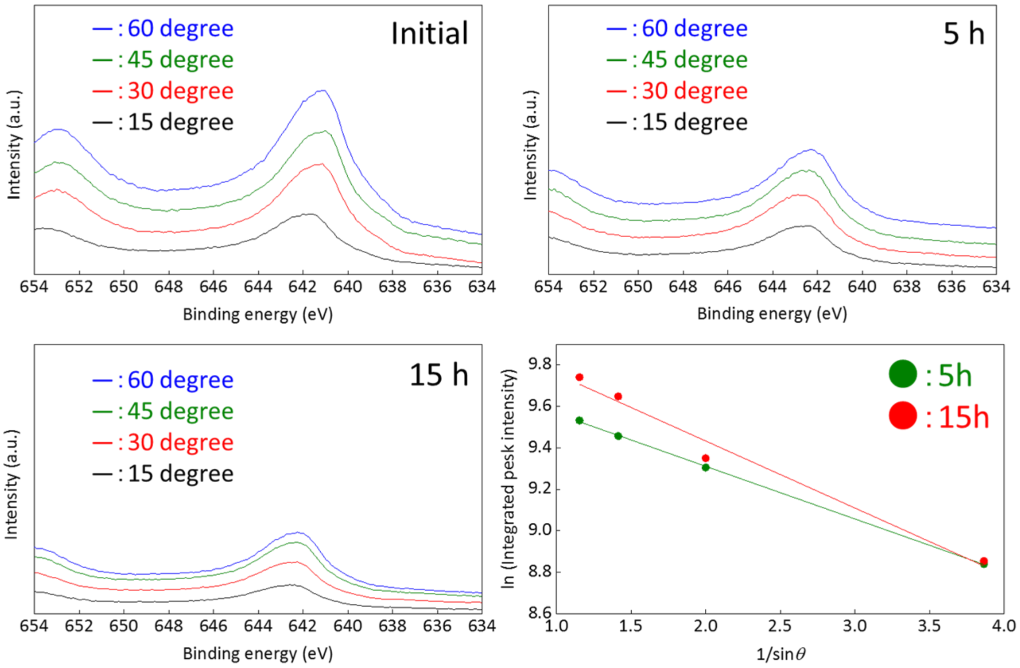

Fig. S3. Angle-resolved XPS-Mn2p core level spectra of the FAS17-SAM-coated $LiNi_{0.5}Mn_{1.5}O_{4-\delta}$ crystals fabricated with different SAM coating times: (a) as prepared (initial), (b) 5h, (c) 10h. (d) The changes in the integrated peak intensity of XPS-Mn2p core level spectra as a function of detection angle. The slope indicated d/λ, where d and λ represent thickness and photoelectron mean free path. The d was evaluated by using semi-empirical equation reported in Ref. 27 [Fadley, C. S.; Baird, R. J.; Siekhaus, W.; Novakov, T.; Bergstrom, S. Å. L. *J. Electron Spec*.**1974**, *4*, 93.]

Table S1. The discharge capacities of the four different cells with different FAS-SAM treatment time under the first three cycles

Treatment time / h	0	5	10	15
1st Discharge capacity ($mAhg^{-1}$)	125.78	124.03	126.21	125.47
2nd Discharge capacity ($mAhg^{-1}$)	125.78	123.69	125.87	125.44
3 rd Discharge capacity ($mAhg^{-1}$)	125.58	123.33	125.76	125.32

Table S2. The changes in the relative atomic concentration of Mn^{3+}/Mn^{4+} as evaluated after 100 cycles at 1 C, as evaluated by XPS

	Mn $2p_{3/2}$ atomic concentration / %			
	initial		After 100 cycles	
Treatment time / h	Mn^{3+}	Mn^{4+}	Mn^{3+}	Mn^{4+}
0	20.17	79.83	16.68	83.32
5	20.15	79.85	18.26	81.74
10	20.16	79.84	18.50	81.50
15	20.19	79.82	18.62	81.38
